# Immersive Surgical Anatomy of the Far-Lateral Approach

**DOI:** 10.7759/cureus.31257

**Published:** 2022-11-08

**Authors:** Andre Payman, Jorge Rios Zermeno, Ankit Hirpara, Ivan H El-Sayed, Adib Abla, Roberto Rodriguez Rubio

**Affiliations:** 1 Neurological Surgery, University of California San Francisco, San Francisco, USA; 2 Neurological Surgery, University of Colorado Anschutz Medical Campus, Aurora, USA; 3 Otolaryngology Head and Neck Surgery, University of California San Francisco, San Francisco, USA

**Keywords:** surgical training, medical education, immersive technologies, volumetric models, surgical neuroanatomy, far lateral approach

## Abstract

The far-lateral (FL) approach is a classic neurosurgical technique that enables access to the craniocervical junction, which includes the lower clivus, the anterior foramen magnum, and the first two cervical vertebrae. The FL approach also provides access to the inferior cranial nerves (i.e., CN IX, CN X, CN XI, and CN XII), distal portions of the vertebral artery (VA), and inferior basilar trunk. Recent advances in three-dimensional (3D) technology as well as dissections allow for a better understanding of the spatial relationships between anatomical landmarks and neurovascular structures encountered during neurosurgical procedures. This study aims to create a collection of volumetric models (VMs) obtained from cadaveric dissections that depict the FL approach's relevant anatomy and surgical techniques. We describe the relevant multilayer anatomy involved in the FL approach and discuss modifications of this approach as well. Five embalmed heads and two dry skulls were used to record and simulate the FL approach. Relevant steps and anatomy of the FL approach were recorded using 3D scanning technology (e.g., photogrammetry and structured light scanning) to construct high-resolution VMs. Images and VMs were generated to demonstrate major anatomical landmarks for the FL approach. The interactive models allow for clear visualization of the surgical anatomy and windows in 3D and extended reality, rendering a closer look at the nuances of the topography experienced in the laboratory. VMs can be valuable resources for surgical planning and anatomical education by accurately depicting important landmarks.

## Introduction

The unilateral suboccipital approach to the lower clivus (LC), the predecessor to the far-lateral approach (FL), was first described in 1972 by Hammon and Kempe [1]. The FL approach was developed and described by Roberto Heros in 1986 and has become the most commonly used approach for lower clival lesions [2,3]. For decades, neurosurgeons have treated lesions in this region using different anterior, lateral, and posterior approaches.

The three main described approaches to the LC are the subtonsillar approach, the endoscopic-endonasal far-medial approach, and the standard and extended variations of the FL approach, each with its advantages and limitations [3]. In particular, the FL approach can be used to treat a variety of pathologies within the LC area, including congenital diseases, vascular lesions, tumors, and degenerative diseases. Using lateral parasagittal and craniocaudal extensions, the FL approach offers dynamic corridors that allow access to peritruncal neurovascular structures located within the inferior anterior half of the posterior fossa, providing higher surgical freedom when compared with more standard posterolateral exposures obtained with retrosigmoid or suboccipital craniotomies. A clear knowledge of the anatomo-functional aspects of the craniocervical junction (CCJ) is pivotal to understanding the FL and its extensions, along with their potential clinical implications. This topic has been previously reviewed in our collection [4,5].

Previous studies have described the technique and quantified the surgical exposure and freedom provided by the FL approach using standard photography and videography methods; however, an analysis of the surgical neuroanatomy with present-day multimedia technology and interactive volumetric models (VMs) that improves our three-dimensional (3D) understanding of the relevant layer-by-layer structures, facilitates our visuospatial understanding of the relevant surgical anatomy, and helps the surgeon perform safe and accurate procedures has not been undertaken. The VMs in this paper demonstrate a faithful 3D representation of the soft tissue and cranial anatomy, surgical landmarks, and techniques of the FL approach to serve as a valuable tool for anatomical education and structural understanding of the posterolateral skull base.

## Technical report

Materials and methods

Five human cadaveric heads, embalmed and injected with colored latex, were prepared for anatomical dissections in a simulated surgical environment in the Skull Base and Cerebrovascular Laboratory at the University of California San Francisco (UCSF) with a surgical microscope (OPMI Pentero 900, Carl Zeiss AG, Oberkochen, Germany). Dissections were documented photographically with a digital single-lens reflect camera (D850, Nikon, Tokyo, Japan) in simulated studio lighting. For macrophotography, a telephoto prime lens (Nikkor 105mm f/1.4E ED, Nikon) and focus stacking software were utilized (Helicon Focus, HeliconSoft Ltd., Kharkov, Ukraine). A dry skull specimen and selected craniocervical and dissected specimens were reconstructed as VMs obtained via surface scanning techniques (i.e., photogrammetry and structured light scanning). The workflow followed by our laboratory is documented in a previously published paper [6]. The polygon count and topology of the VMs generated from the scans were postprocessed and optimized for online visualization using computer graphics software (Zbrush 2021.5.1, Pixology Inc., Los Angeles, CA, USA, and Blender 3.3, Blender Foundation, Amsterdam, Holland). Additional nonscanned VMs were digitally sculpted using the same computer graphics software. Corresponding physically based rendering texture maps were generated via procedural and photo projection methods with a 3D texturing program after creating individual UV maps (Substance Painter, Adobe, San Jose). 

No IRB/ethics committee approval was required for this study as it does not involve any private health information.

Virtual platform

The anatomical VMs were uploaded to a web-based 3D model viewer (Sketchfab, Sketchfab Inc., New York, NY, USA), a platform that belongs to a series of new modalities meant to enhance the immersive and functional capacities of the VMs. Once the VMs were uploaded, the virtual scene was prepared for its real-time rendering. Position, lighting, materials, and filters were set to highlight the regions of anatomical interest. Strategic points were labeled and annotated for an interactive experience. Views of the models were set for both 2D and 3D experiences. The stereoscopic version of the virtual scene was set up and tested using a virtual reality (VR) headset (HTC Vive, HTC Co., Taiwan, China) and a browser compatible with WebVR technology (Firefox Nightly, Mozilla Co., Mountain View, CA) [6].

The following instructions can be used to manipulate all VMs: to move, use the left click and drag; to zoom in and out, use the mouse scroll; to view annotations, click the numbers; and to move around the object, tap or press trigger on the floor using the blinking yellow circle as a pointer. For mobile augmented reality (AR), click the AR icon (cube) in the top right corner and aim at a horizontal flat surface; when the surface is detected, tap on it to place the VM. For smartphones and VR-ready computers, click the glasses icon “view in VR”; Google Cardboard and YouTube mobile apps are needed to view the video in VR mode. First, open the video on the YouTube mobile app and tap the Cardboard icon. Next, place the mobile device inside the Google Cardboard. Finally, look around to view the video in 360 degrees. The quality of textures and navigation style can be modified by clicking the gear (Settings) icon [5].

Anatomical description

The following sections review the relevant anatomical structures of the FL approach, including the bony, myofascial, and extracranial neurovascular anatomy. The soft tissue of the posterolateral region of the skull is also described layer by layer, from superficial to deep, superior to inferior, and lateral to medial. Lastly, the important bony landmarks for performing the craniotomy and condylar resection are described.

*Bony Anatomy* 

A comprehensive appreciation of the bony anatomy and natural boundaries is essential to identify important landmarks to successfully access the lower clivus (LC) through the corridor of exposure provided by the FL approach. The exposure is limited anterolaterally by the sigmoid sinus, superiorly by the superior nuchal line (SNL) and the transverse sinus, inferiorly by the C3/4 vertebrae and posteromedially by the median nuchal line. Furthermore, a thorough understanding of the CCJ’s posterior anatomy is essential to perform the FL approach successfully [5].

One of the first landmarks identifiable during the FL approach is the SNL on the squamous region of the occipital bone. The SNL serves as a landmark for the transverse sinus and provides attachments for the most superficial muscles dissected in the FL approach. These include the trapezius medially, the medial portion of the sternocleidomastoid (SCM), and the splenius capitis; the semispinalis capitis lies in a slightly deeper layer. The inion is the tip of the external occipital protuberance, lying medially on the same level as the SNL. The inferior nuchal line (INL), located caudal to the SNL, provides attachments to some of the deeper muscles detached in the FL approach. Between the INL and the SNL, the semispinalis capitis attaches medially, and the obliquus capitis superior attaches laterally. Inferior to the INL, the bone provides attachments for the rectus capitis posterior minor and major, medially and laterally, respectively.

**Figure 1:**
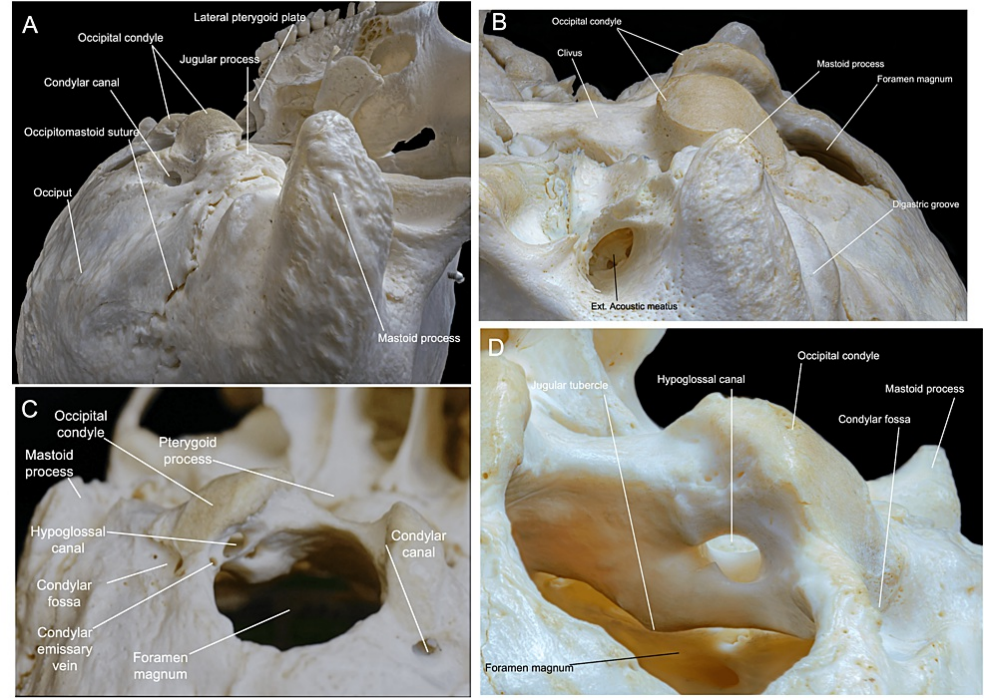
Occipital bone anatomy. (A) Superolateral perspective of the inferior view of the skull showing the bony prominences, such as the mastoid tip and the condyles. (B) Inferior and lateral view of the skull illustrating the same structures as the previous and the digastric groove. (C) Inferior and posterior perspective of the occipital bone where we can observe the hypoglossal canal, condylar canal and fossa, and emissary canal. (D) Close-up perspective of the occipital condyles and foramen magnum, appreciating in detail the hypoglossal canal and jugular tubercle. (Published with permission of the University of California San Francisco’s Skull Base & Cerebrovascular Laboratory.)

**Figure 2:**
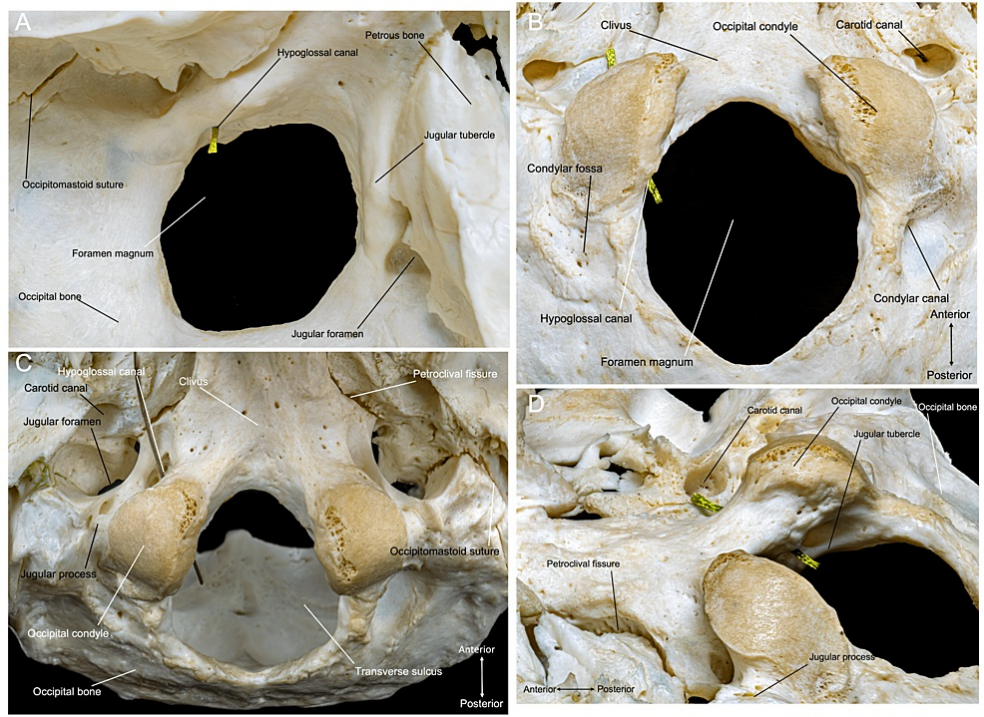
Occipital bone overview. (A) Superior perspective of the occipital bone, depicting the jugular foramen, jugular tubercle, lower clivus, and foramen magnum. (B) Inferior perspective of the skull, where we appreciate the direction of the hypoglossal canal in relation to the direction of the occipital condyle. (C) Anterior and inferior perspectives of the skull, showing the extracranial jugular foramen and condyles. (D) Superolateral perspective of the occipital condyles and the hypoglossal canal. (Published with permission of the University of California San Francisco’s Skull Base & Cerebrovascular Laboratory.)

The lateral regions of the occipital bone are located on the either side of the foramen magnum. The lateral regions contain the occipital condyles, jugular processes, and jugular notches (Figures 1C, 2B). We further describe the anatomy of the condyles in detail in the Discussion (see the Modifications of the Standard FL Approach section). 

**Figure 3:**
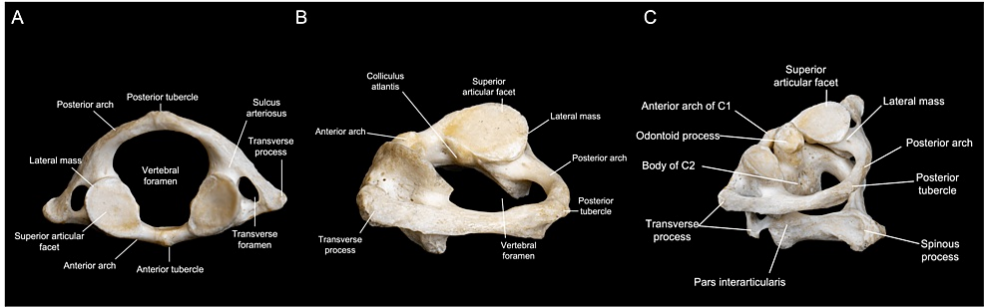
C1 anatomy. (A) Superior view of the C1 vertebra, showing the transverse foramina, medular canal, and articular facets. (B) Surgical perspective of C1 during a left FL approach. (C) Superior and lateral view of articulated C1 and C2, revealing the relationship between the odontoid process and the anterior arch of C1. (Published with permission of the University of California San Francisco’s Skull Base & Cerebrovascular Laboratory.) FL, far lateral

The first cervical vertebra is called the atlas and contains notable landmarks, including the sulcus arteriosus, transverse foramen, transverse process, and posterior tubercle (Figure 3). The vertebral artery (VA) travels through the transverse foramen from C6 to C1 and then rests on the sulcus arteriosus on the posterior arch of C1. The transverse process serves as an attachment to the obliquus capitis superior and inferior, whereas the posterior tubercle serves as an attachment site to the rectus capitis minor (Figure 3A).

The second cervical vertebra, the axis, is located below the atlas. The spinous process of C2, located in the medial posterior portion of the bone, attaches to the origins of the rectus capitis posterior major and the obliquus capitis inferior (Interactive Model 1). This vertebra comprises the inferior limit of laminectomy for the standard FL approach.  

**Video 1:** Volumetric model depicting muscular attachments within the suboccipital region.

Myofascial Anatomy

Superficially in the posterolateral region for the FL approach, the most lateral muscle found is the SCM. The SCM inserts on the lateral surface of the mastoid and the lateral half of the SNL, reaching the upper part of the anterior surface of the manubrium (the sternal head) and the medial third of the clavicle (the clavicular head). The trapezius muscle is located medially to the SCM; it originates from the SNL, the external occipital protuberance, and the nuchal ligament and follows a lateral direction to the scapula.

The splenius capitis is located most laterally in the second layer of muscles. The splenius capitis travels in an oblique direction and inserts on the lateral portion of the mastoid and the lower half of the nuchal ligament of the superior nuchal line. The splenius capitis is located underneath the mastoid portion of the SCM. Parasagittal to the midline, the semispinalis capitis inserts between the superior and INLs. The lateral insertion of the semispinalis capitis is partially covered by the medial insertion of the splenius capitis, while the superior portion of the trapezius covers the medial insertion. In the third layer, beneath the splenius capitis, the longissimus capitis inserts laterally at the posterior margin of the mastoid process, lying slightly lateral and superficial to the posterior belly of the digastric muscle, which inserts on the digastric groove over the inferior surface of the skull, located medially to the mastoid process. The posterior belly of the digastric muscle is located lateral to the trunk of the OA, traveling over the occipital groove (Interactive Model 2). 

**Video 2:** Volumetric model showing the superficial muscular layers of the suboccipital region.

**Figure 4:**
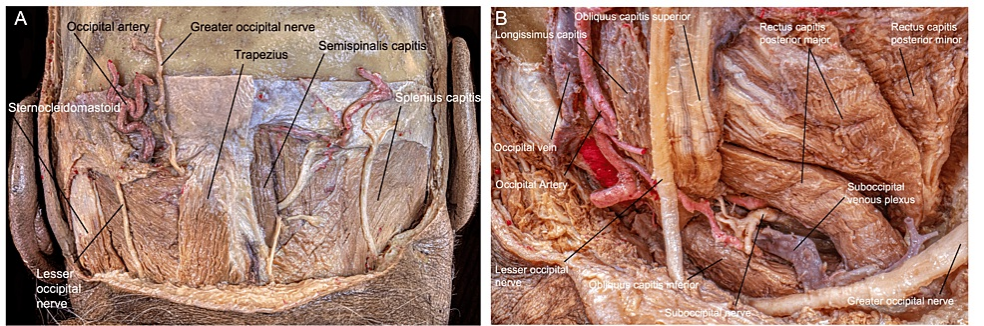
Myofascial anatomy during the FL approach. (A) Posterior view of the muscular, vascular, and nervous anatomy encountered during the FL approach. (B) Close-up perspective of the left suboccipital muscles. We can appreciate the occipital artery, suboccipital plexus, and GON and LON. (Published with permission of the University of California San Francisco’s Skull Base & Cerebrovascular Laboratory.) FL, far lateral; GON, greater occipital nerve; LON, lesser occipital nerve

The fourth layer, or suboccipital muscles group, lies beneath the semispinalis capitis, as deeper muscles of the posterior neck (Interactive Model 3). This muscular group comprises four muscles: obliquus capitis superior, obliquus capitis inferior, rectus capitis posterior major, and rectus capitis posterior minor. The obliquus capitis superior originates in the transverse process of the C1 vertebra and inserts between the superior and INLs underneath the lateral portion of the semispinalis capitis. The rectus capitis posterior major originates in the spinous process of the C2 vertebra, and it inserts on the lateral half of the INL beneath the obliquus capitis superior. The rectus capitis posterior minor originates in the posterior tubercle of the C1 vertebra and inserts into the medial half of the inferior nuchal line. The obliquus capitis inferior also originates on the spinous process of the C2 vertebra, and it inserts on the transverse process of the C1 vertebra (Figure 4A).

**Video 3:** Volumetric model showing the suboccipital muscular group and relevant neurovascular structures.

Insertion points of all the suboccipital muscles, excluding the rectus capitis posterior minor, form a triangular window named the suboccipital triangle, which contains structures of the horizontal portion of the VA, suboccipital venous plexus, and suboccipital nerve and the posterolateral portion of the posterior arch of C1. The following section discusses the relationships between the VA and OA.

Finally, the fifth muscle layer includes the rectus capitis lateralis, which originates from the transverse process of the atlas and inserts onto the external surface of the jugular process of the occipital bone. The identification and dissection of this muscle are crucial for the paracondylar variation of the FL approach. A summary of the main muscles within the suboccipital region can be found in Table 1.

**Table 1:** Main muscular attachments of the suboccipital region. C, cervical; CN, cranial nerve; O, oblique; PS, parasagittal; INL, inferior nuchal line; SNL, superior nuchal line

Location	Muscle	Origin	Insertion	Innervation	Direction of muscle fibers (function)
First layer	Trapezius muscle	SNL	Clavicle and scapula	CN XI	O (Scapulary movement and spinal stabilization)
	Splenius capitis muscle		Mastoid process and inferior to lateral third of SNL	C3 and C4 nerves	O (Lateral head flexion and rotation)
Second layer	Semispinalis capitis muscle		Between SNL and INL	Greater occipital nerve (C2)	PS (Bilateral extension and horizontal rotation of head)
Third layer	Longissimus capitis muscle		Posterior medial mastoid process	Dorsal rami of spinal nerves	PS (Extension and rotation of the head)
	Posterior belly of digastric muscle	Digastric groove		CN VII	PS (Elevation of the hyoid bone)
Fourth layer (suboccipital muscles)	Rectus capitis posterior major muscle	Spinous process of C2	Lateral of INL	Suboccipital nerve (C1)	PS slightly O (Extension and rotation of atlantooccipital joint)
	Rectus capitis posterior minor muscle	Posterior arch of C1	Medial part of INL	Suboccipital nerve (C1)	PS slightly O (Backward extension of the head)
	Obliquus capitis superior muscle	Transverse process of C1	Lateral half of INL	Suboccipital nerve (C1)	O (Head extension and flexion of the head to the ipsilateral side)
	Obliquus capitis inferior muscle	Spinous process of C2	Transverse process of C1	Suboccipital nerve (C1)	O (Rotation of head and C1 vertebra through atlantoaxial joint)
Fifth layer	Rectus capitis lateralis muscle	Transverse process of C1	Jugular process	C1 and C2 nerves	PS (Stabilization of the atlantooccipital joint)

Neurovascular Anatomy

A precise knowledge of the 3D arterial networks involved in the blood supply of specific regions of soft tissue, also known as angiosomes, can allow for better planning of the skin incision, a safer dissection at multiple stages of the approach, and ultimately a more rapid wound recovery by preserving valuable superficial vascular networks. Relevant to the FL, the occipital, transverse cervical, deep cervical, posterior auricular, and branches of the VA supply angiosomes of the posterolateral head and neck region. In this section, we will discuss the main vascular components found during the FL approach and their relevance at different stages of the exposure.

Vertebral artery: The VA typically arises from the subclavian artery and travels cranially through the transverse foramina of the cervical spine to supply various structures at the posterior region of the skull base, including the brainstem and the cerebellum. Traditionally, the VA is divided into four segments, namely, V1 to V4. The first segment (V1), known as the preforaminal segment, arises dorsally from the posterosuperior surface of the subclavian artery and conventionally enters the transverse foramen of the C6 vertebra. Occasionally, this segment may enter the transverse foramina of C7 or C5. The V2 segment, the foraminal segment, travels within the transverse foramina from C6 to C2, after which it makes a 45-degree lateral turn and passes through the foramen of C1. The V3 segment, named the atlantic segment, is a short and tortuous segment that makes a posteromedial turn around the superior articular process of C1 and runs horizontally along a groove of the posterior arch of the atlas, called the sulcus arteriosus. Notably, the midline and the mastoid tip serve as markers to locate the lateral portion of the V3 segment. The V3 segment then runs posteriorly to the posterior atlantooccipital membrane, after which it enters the foramen magnum and pierces the dura mater. Occasionally, a bony arch, known as the ponticulus posticus, will bridge the sulcus arteriosus. This osseous variation results from the ossification of the atlantooccipital ligament and causes a complete enclosure of the V3 segment. It is important to bear in mind this potential ossification while planning an FL approach as the overall prevalence of this anatomical variation is approximately 16.9%. The intradural V4 segment is entirely intracranial and terminates when the vertebral arteries join to form the basilar artery [7].

The V3 segment of the VA is most relevant for the FL approach, and its anatomy must be appreciated to perform a successful surgery. The course of the V3 segment can be divided into two portions: a vertical portion (V3V) that travels from the transverse foramen of C2 to C1 and a horizontal portion (V3H) that travels in the sulcus arteriosus and up to the dura mater. The V3H portion exits the transverse foramen of C1 and immediately turns to travel transversely above the C1 posterior arch, with part of its course in the sulcus arteriosus. As it reaches the medial portion of the lateral mass of the atlas, it changes direction from transverse to slightly oblique. The distal portion runs superomedially to the atlantooccipital joint, entering the dura at the lateral margin of the foramen magnum [7]. Notably, V3 is the most mobile segment of the VA, allowing for stretching and compression according to the head position. The V3 segment has one or more muscular branches coursing posteriorly at the upper exit of the C1 transverse foramen and a posteromedial branch at the junction of the horizontal and oblique portions of the V3 segment. Distal to these branches, the V3 occasionally has a posterior meningeal artery branch, which may originate at the beginning of the V4 segment. A final variation to consider is an extradural origin of the posteroinferior cerebellar artery (i.e., V3H), which conventionally branches out intradurally from V4.

Occipital artery: The occipital artery (OA) arises from the posterior or lateral aspect of the external carotid artery (ECA), adjacent to the origin of the facial artery. The OA provides arterial supply to most of the muscles of the posterior and lateral upper portion of the neck, the muscles of the suboccipital region, and the occipital scalp. The OA is divided into three main segments: ascending cervical, cervico-occipital or horizontal, and ascending occipital.

In the ascending cervical segment, the OA runs in a posterosuperior direction from its origin, along the medial surface of the posterior belly of the digastric muscle. It extends to the occipital groove of the mastoid process, covered by the anterior surface of the longissimus capitis. The ascending cervical segment has multiple branches that supply various muscles in the posterior neck and the skull base. After emerging from the occipital groove, the cervico-occipital segment of the OA is covered by the splenius capitis. It then turns medially and slightly superiorly, passing the obliquus capitis superior and semispinalis capitis, and then piercing the insertion of the splenius capitis at the SNL. There are two main branches of the cervico-occipital segment of the OA, a superficial descending branch and a deep descending branch. These branches are critical as a source of communication between the VA and the ECA systems. Additionally, one to three cervico-occipital branches arise from the OA and run parallel to it, supplying the SCM and splenius capitis muscles; one to three transosseous branches of the cervico-occipital segment supply the dura of the posterior fossa and the mastoid and occipital bone. Occasionally, one to two branches supply the occipital condyle and the occipitocervical junction.

After intersecting the SNL, the ascending occipital segment of the OA begins. It continues over the occipital muscle surface, perfusing the muscle and the occipital scalp. At about 5 cm above the SNL and 2-4 cm laterally to the midline, the OA divides into two terminal branches that ascend almost parallel to each other. The distal branches form an extensive anastomotic network with branches of the posterior auricular, superficial temporal arteries, and the contralateral OA [8]. This rete is especially important when planning the superior and lateral portions of the incision; if the boundaries are exceeded, the trunk may be compromised.

Other distal branches of the OA worth mentioning include a superficial branch that travels up to the obelion through the parietal foramen and irrigates the posterior dural convexity, branches to the endolymphatic duct and sac, and anastomoses between VA and OA branches (i.e., radicular artery of C2 and muscular branches of V3H) [8].

**Figure 5:**
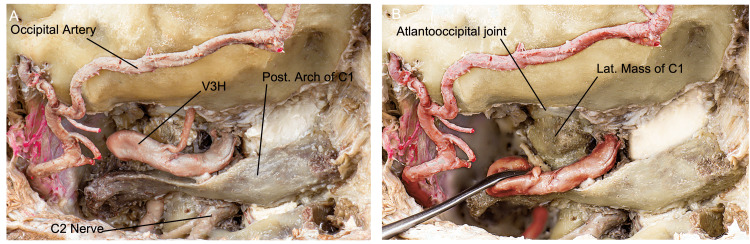
Overview of the craniocervical junction anatomy relevant to the FL approach after muscle dissection and suboccipital plexus resection. (A) Posterior and slightly lateral perspective of a left approach, with (B) emphasis on the occipital condyle–C1 articulation. (Published with permission of the University of California San Francisco’s Skull Base & Cerebrovascular Laboratory.) C, cervical; FL, far lateral; V3H, vertebral artery horizontal segment

Dural supply: A branch of V3H, the posterior meningeal artery, supplies the dura over the posterior atlantooccipital space, the falx cerebelli, and the medial and paramedial cerebellar fossa (Figure 5A). Anterolaterally, the neuromeningeal trunk of the ascending pharyngeal artery supplies the dura of the LC, the inferomedial petrosal surface, and the inferior cranial nerves and foramina via the clival, jugular, and hypoglossal branches. The mastoid branch of the occipital artery supplies the dura over the lateral portion of the posterior petrosal surface, including the posterior edge of the jugular foramen (JF) and the lateral and paramedial cerebellar fossa. Furthermore, the petrosquamosal branch of the posterior division of the middle meningeal artery supplies the dura around the confluence between the superior petrosal, transverse, and sigmoid sinus, and the superior portion of the dura of the lateral posterior fossa [9]. The location and role of these dural suppliers from extracranial arteries play a major role while tailoring an FL exposure to deal with certain vascular malformations and tumors of the region.

Venous Structures 

The venous structures in the suboccipital region include the suboccipital venous plexus, the venous compartment cushioning the V3H portion, the VA venous plexus around the V3V segment, and the vertebral venous plexus related to the spine. The suboccipital venous plexus is located between the second and fourth muscle layers, lying deep to the semispinalis capitis and splenius capitis muscles. The suboccipital venous plexus communicates with the venous compartment cushioning the V3H portion, the VA venous plexus, the vertebral venous plexus, and the transverse sigmoid sinus through the mastoid emissary and occipital veins. The suboccipital venous plexus drains into the transverse sigmoid sinus junction via the mastoid emissary and occipital veins [10]. The occipital vein drains deep CCJ structures, and it serves as a connection between intracranial veins and superficial veins in the region through the mastoid emissary vein.

The anatomy of the suboccipital complex is strikingly analogous to that of the cavernous sinus, which is why it is often referred to as the suboccipital cavernous sinus. Some of the shared structural similarities reside in the venous cushioning, surrounding nerves, skull base location, and anatomical properties of V3 and the petrous-cavernous ICA, especially their loops, branches, fibrous rings, and periarterial autonomic neural plexus.

The venous compartment within the suboccipital complex communicates with the occipital sinus through the marginal sinus and with the contralateral sinus through the vertebral venous plexus. It also communicates with the jugular bulb and vein through (1) the anterior condylar vein alongside the hypoglossal nerve and within the hypoglossal canal; (2) the posterior condylar vein located posterior and superior to the occipital condyle; (3) the lateral condylar vein, which is lateral to the occipital condyle and connects the jugular vein with the venous compartment cushioning the V3H portion; and (4) the suboccipital venous plexus [10].

Nerves

The greater occipital nerve (GON) is a purely afferent nerve that emerges from the dorsal ramus of C2 and has branches that follow collateral branches of the OA to the occipital muscle traveling upward parallel to the cervicooccipital segment of the OA [8]. Specifically, it rises past the suboccipital triangle posteriorly and between the obliquus inferior and semispinalis capitis, piercing the latter muscle and the trapezius. As it continues to rise, it passes the SNL and intersects with the OA at the medial edge of the SCM muscle attachment. The GON then innervates the skin of the nuchal region and the semispinalis capitis.

The lesser occipital nerve (LON), which emerges from the ventral rami of C2 and C3, is notable in the FL approach. It runs lateral to the GON and inferior to the splenius capitis muscle. The LON ascends along the posterior border of the SCM muscle, sending anterior branches to the ear and posterior branches to the mastoid area. The posterior branches overlap with branches of the GON, whereas the inferior branches overlap with branches of the great auricular nerve.

The suboccipital nerve is another significant nerve in the FL approach. It rises from the dorsal ramus of C1 and travels through the sulcus arteriosus alongside the VA. The posterior occipitoatlantal ligament can be found within this groove space. It then travels into the suboccipital triangle, innervates the suboccipital muscles, and sends somatic motor fibers to the hypoglossal nerve. The suboccipital nerve courses inferiorly to the V3H segment of the VA and superiorly to the posterior arch of the atlas. It branches from its dorsal ramus to a medial ramus for the rectus capitis posterior major and minor muscles, a lateral ramus for the obliquus capitis inferior, and an inferior ramus for the obliquus capitis superior (Figure 5A). 

Surgical technique

Positioning 

The FL approach may be used with the patient in a modified park bench, also known as the three-quarter prone, or in the semi-sitting position. The semi-sitting position requires the main body of the operative table to be in the Trendelenburg position and the legs to be elevated. The headboard should be tilted up to a maximum of 60 degrees [11]. The modified park bench position requires the patient to be placed so the lesion side faces upward. The operating table is extended by placing a board under the mattress and pulling the mattress and board beyond the edge of the table [12]. This creates a gap between the head holder and its attachment to the table, allowing the dependent arm to hang over the end of the table. This also permits effective head rotation into an ideal position and minimizes brachial plexus compression, improving venous return compared to the full prone position. There should be head flexion until the chin is one finger’s breadth (2 cm) away from the sternum, and 45 degrees rotation away from the side of the lesion, followed by lateral flexion 30 degrees down toward the floor [12]. A Mayfield head holder is recommended, and the pins should be placed on both sides on the superior temporal line. Care should be taken to avoid important neurovascular structures - notably the superficial temporal artery, OA, and posterior auricular artery with their associated nerves - and placing them through the temporal muscles or on delicate squamous parts of the temporal bone [11]. The mastoid process is conventionally set as the highest point in the operative field. The superior facing shoulder of the patient is then taped to keep the cervical-suboccipital angle open [12].

Incision of the Skin

Understanding the scalp soft tissue and vascular anatomy is essential to avoid skin ischemia, muscle atrophy, dysesthesia, or scar neuroma after an FL approach. The dissection of the soft tissues should prioritize the preservation of extracranial neurovasculature, extracranial sensory nerves, and myofascial structures. The common variations of skin incisions for the FL approach include the horseshoe, the hockey stick, and C-shaped incisions [4,11]. The hair should be shaved just before surgery, treated with ether-soaked gauze, and the incision line should be marked. The inion, asterion, spinous process of C2 (for the horseshoe incision) or C4 (for the hockey stick incision) and mastoid apex should be marked; then, they should be linked to form the full incision path. The incision begins in the cervical midline over the C4 spinous process, extending superiorly to the inion, then coursing laterally along the SNL to the mastoid bone, and finishing inferiorly at the mastoid tip [12]. Bipolar coagulation can help avoid scalp artery bleeding. Wet gauze can be placed while applying traction to the scalp flap to spare the use of hemostatic clips. Abundant irrigation of the operative field with physiological solution is imperative to avoid gaseous embolization while dissecting in the vicinity of the vertebral venous plexus and emissary condylar and mastoid veins (Interactive Model 4) [11].

**Video 4:** Volumetric model showing the relevant angiosomes (right side) and landmarks for skin incisions use in the far lateral approach (left side).

Dissection of the Muscles

We now describe the muscle dissection, beginning superiorly and superficial, moving inferiorly and medially, and finishing deep, inferiorly and laterally. Depending on the lowest end of the skin incision, the scalp flap could be reflected downward medially or laterally, showing the most superficial muscle layers formed by the SCM and trapezius. A parallel cut just below the SNL should be made, leaving a 1-cm cuff of fascia to reattach the muscle during closure. This cut extends laterally to the mastoid bone and inferiorly to its tip [12]. The trapezius should be dissected and reflected medially and inferiorly, and the SCM should be divided and reflected laterally, exposing the upper extension of the semispinalis capitis and splenius capitis. Both muscles should be detached and reflected medially and inferiorly to expose medially the superior attachments of the rectus capitis posterior minor and major muscles and laterally the longissimus capitis muscle, the deep lamina of deep cervical fascia, and the OA. The longissimus capitis can then be detached from the posteromedial edge of the mastoid process and reflected downward to expose the superior and inferior oblique muscles and the transverse process of the atlas [4]. Reflecting these muscles medially is preferred to laterally and inferiorly because it avoids interference of the muscle flap with the hands of the surgeon. After reaching the occipital triangle, the VA should be identified, as well as the nerve roots of C1 and C2. The medial insertions of the superior and inferior oblique muscles should then be detached and reflected medially. The VA should be dissected carefully by first localizing its medial margin with the aid of doppler and conducting a blunt dissection along the posterior margin of the sulcus arteriosus of C1 to finally expose the entrance of the VA into the dura (Interactive Model 5) [11].

**Video 5:** Volumetric model depicting muscular attachments within the suboccipital region.

It is possible to expose the VA using a plane-by-plane dissection of (1) the SCM and trapezius muscles, (2) the splenius capitis muscle, (3) the semispinalis capitis and longissimus capitis muscles, and (4) the rectus capitis posterior minor, rectus capitis posterior major, inferior oblique, and superior oblique muscles. However, this approach increases the risk of necrosis and devascularization of the flap. Instead, the superior and INLs can be used to separate the muscles. After the incision, the flap is subperiosteally lowered to the INL, allowing the first three muscle planes to remain attached to the myocutaneous flap and the fourth plane to the skull base. Afterward, the suboccipital triangle, the best landmark for V3, can be easily exposed [13]. Specifically, V3H can be found within the C1 vertebral groove once the superior oblique muscle is separated from the fat tissues and paravertebral venous plexus and toward the C1 transverse process if further lateral exposure is needed [14].

In terms of morphometrics, the average distance between the superior edge of V3H and the inferior edge of the occipital bone is 6 mm. The medial edge of V3H can be found at about 15 mm from the midline [15]. Lastly, the mean distance between the mastoid tip and the lateral edge of V3H is approximately 21 mm [15].

**Figure 6:**
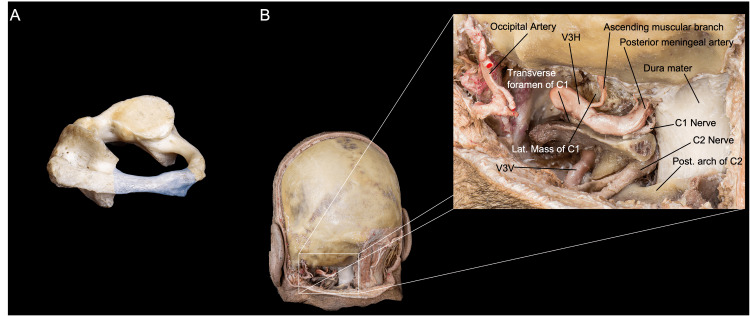
Overview of the resection of the posterior arch of C1. (A) We observe shaded in blue the portion of the posterior arch to be resected. (B) Posterior and close-up perspective of the approach after removal of the posterior arch of C1. (Published with permission of the University of California San Francisco’s Skull Base & Cerebrovascular Laboratory.) C, cervical; V3V, vertebral artery vertical segment; V3H, vertebral artery horizontal segment

The bone removal begins with the removal of the mid portion of the posterior arch of C1, with cuts made in a rostral to caudal direction to protect the VA. Additional bone can be removed laterally to the transverse foramen to release and mobilize V3 if necessary. The posterior atlantooccipital membrane, continuous inferiorly with the ligamenta flava, should be detached from the posterior margin of the FM. Dural adhesions, including the posterior atlantooccipital membrane and the dura around the FM, should be stripped, and the lip of the FM should be used as the epidural access for the drill (Figure 6B) [12].

**Figure 7:**
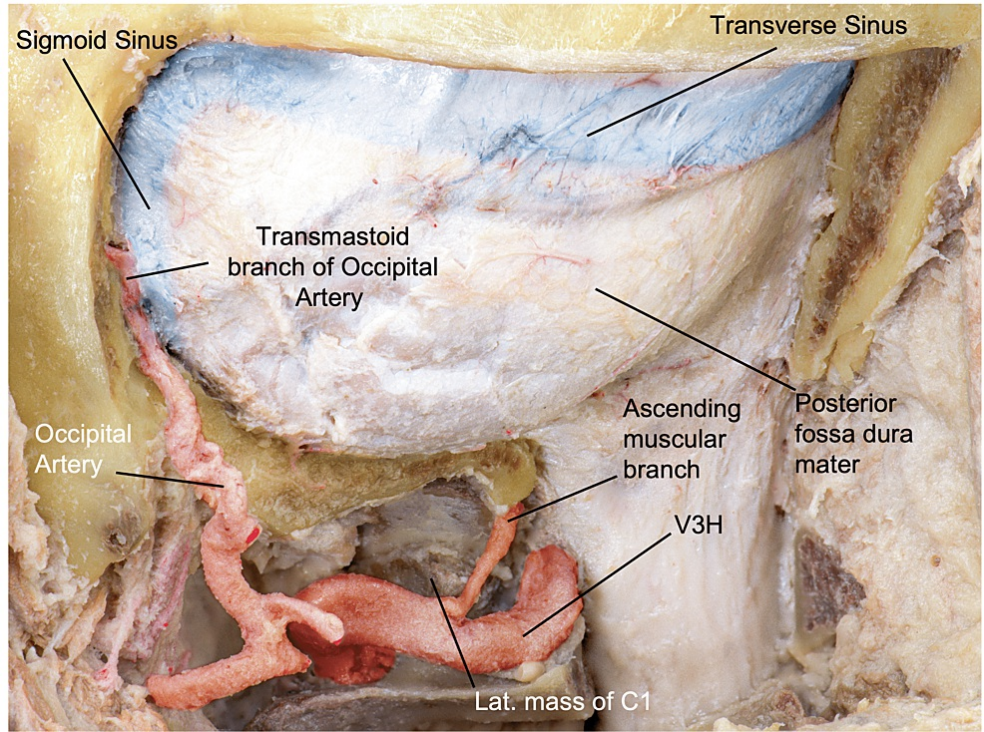
Surgical view after craniotomy, showing the dura mater of the posterior fossa and the spine, the occipital and vertebral arteries, and the transverse and sigmoid sinuses. (Published with permission of the University of California San Francisco’s Skull Base & Cerebrovascular Laboratory.) C, cervical; V3H, vertebral artery horizontal segment

Burr holes should be made in the lower portion of the occipital bone, one medial and one lateral close to the mastoid. Two more should be made near the inion medially, and two more by the asterion at the superior and inferior edges of the transverse sinus. These points should be connected to complete the lateral suboccipital craniotomy [11]. Laterally, the limit should be the posterior edge sigmoid sinus, and superiorly, the limit should be the inferior edge of the transverse sinus. Further mastoid drilling can be performed to expose the entire sigmoid sinus if the retrosigmoid dissection demands more lateral maneuverability. The final step of bone removal is the condylectomy (Figure 7). We will discuss in detail this procedure and its variants in the Discussion section [12].

Dural Opening 

The dural incision should curve from the cervical midline across the marginal sinus to the lateral edge of the craniotomy. An inferior dural incision laterally under C1 mobilizes the flap further laterally against the margin of the craniotomy. The condylectomy is sufficient if no bony prominence obstructs the view of the lateral medulla [12]. The intradural portion of the VA (i.e., V4) is exposed upon dural opening. As the VA pierces the dura, it is enclosed within a fibrous tunnel that attaches it to the posterior spinal artery, dentate ligament, first cervical nerve, and spinal accessory nerve. The posterior spinal artery, therefore, must be preserved due to its relationship with the dural cuff around the VA (Interactive Model 6).

**Video 6:** Volumetric model showing a transcondylar FL approach after dural opening. FL, far lateral

## Discussion

Modifications of the standard FL approach

The FL approach allows for various modified approaches, including the transcondylar, supracondylar, and paracondylar, that permit different visualizations in the LC region for optimal treatment of lesions. In summary, the transcondylar approach proceeds through the occipital condyle and provides access to the LC and premedullary regions. The supracondylar approach proceeds through the area superior to the occipital condyle and provides access to the hypoglossal canal (HC), jugular tubercle (JT), and inferior petroclival junction. Lastly, the paracondylar approach proceeds through the quadrangular area lateral to the occipital condyle (i.e., the jugular process). It provides access to the posterior part of the jugular foramen and the extratemporal segment of the facial nerve (Interactive Model 7).

**Video 7:** Volumetric model depicting craniocervical neurovascular structures and lateral triangles of the posterior fossa (i.e., inferior, central inferior, and central superior) involved in the main modifications of the FL approach (low poly right side). FL, far lateral

We believe that understanding the anatomy of the occipital condyle is essential to comprehend the FL approach and its modifications. In the following sections, we will discuss the morphological characteristics of the occipital condyle.

Relevant Condylar Anatomy for Modifications to the FL

The condyles are paired oval structures located at the base of the occipital bone that articulate inferiorly with the superior facets of the C1 vertebra to form the atlantooccipital joint. The articular surfaces of the condyles are convex and face inferiorly and laterally. Their anterior segments are directed forward and medially, closer together than the posterior segments. The posterior segments extend to the middle of the FM, meaning that the condyles lie within the anterior half of the FM. The medial surface of each condyle has a tubercle for the alar ligament, which connects inferiorly to the odontoid process of the C2 vertebra. The condylar fossa is frequently the site of the posterior condylar canal, which transmits the posterior condylar emissary vein. This canal is directed slightly upward as it proceeds anteriorly to join the sigmoid sinus immediately proximal to where the sinus empties into the jugular bulb. The posterior condylar canal passes superiorly to and usually does not communicate with the anterior condylar canal, also known as the HC [16].

The HC is located within the superior half of the occipital condyle, between the middle and posterior thirds, running obliquely from posteromedial to anterolateral in the axial plane. The intracranial end of the HC is approximately 5 mm superior to the inferior condylar surface and about 5 mm inferior to the JT. The average distance between the posteromedial edge of the condyle and the posterior border of the intracranial end of the HC is 12 mm. In contrast, the length between the extracranial edge and the posterolateral border of the condyle is about 15 mm. The condyle is about 21 mm long on its long axis, meaning that the posteromedial portion of the HC lies at the junction between the middle and posterior thirds of the condyle [4]. Specifically, the canal makes a 49-degree angle relative to the sagittal plane, from medial to lateral. The internal orifice of the canal has an average transverse diameter of 7.4 mm and a vertical diameter of 4.4 mm. The external orifice has an average transverse diameter of 6.1 mm and a vertical diameter of 3.9 mm [17]. The presence of a septate HC, prevalent in about 30% of individuals, should be assessed preoperatively to properly understand the location of the most posterior portion of the CN XII while performing a transcondylar or supracondylar drilling [18]. Lateral and superior to the posterior half of the condyles, a quadrilateral bony projection, the jugular process, articulates with the inferior surface of the petrous portion of the temporal bone. The posterior aspect of the jugular foramen is formed by the jugular notch, which is a groove along the anterior margin of each jugular process.

Occipital Condyle Anatomical Variants 

The extent of bony removal of the occipital condyle for optimal exposure in the far lateral approach and its modifications should be considered before the procedure, and its functional implications should be weighted. Relevant characteristics such as the shape, length, width, and location of the HC should be taken into account.

Among the eight described shapes of the occipital condyle, the oval-shaped is the most commonly found. There are also three high-risk variants for CCJ instability to consider - the triangular, reniform (kidney), and irregular shapes. In general terms, the condyle is classified as short if less than 2 cm in length, moderate between 2 and 2.4 cm in length, and long if greater than 2.4 cm; only the short condyle has relevance regarding CCJ instability. The HC follows a medial to lateral and posterior to anterior direction; it is bordered superiorly by the JT and inferiorly by the occipital condyle. Distances from the posterior edge of the condyle to the HC have been mentioned in the previous section [18].

Transcondylar approach

**Figure 8:**
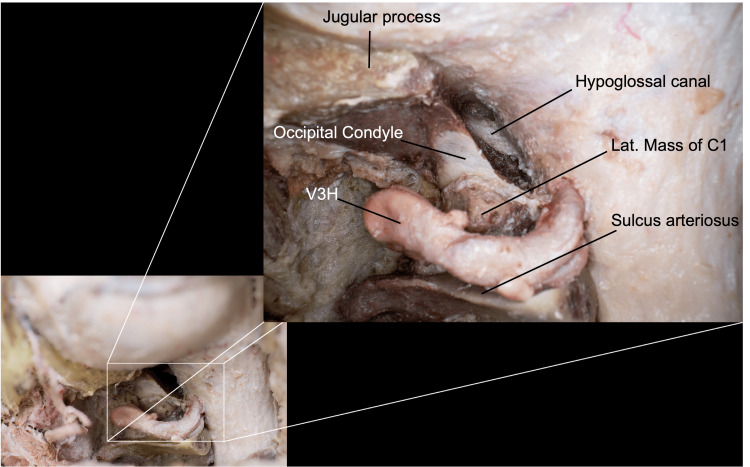
Close-up view after a posterior-third condylectomy (transcondylar approach), where we can observe the hypoglossal canal as our medial limit. Here, we preserved the C0-C1 junction. (Published with permission of the University of California San Francisco’s Skull Base & Cerebrovascular Laboratory.) C, cervical; V3H, vertebral artery horizontal segment

The microsurgical transcondylar approach is directed through the posterior portion of the occipital condyle or atlantooccipital joint and allows for a more direct approach to the LC and anterolateral perimedullary area. A key element of the extradural exposure for this approach is the identification and dissection of V3H, which could be extended laterally to the transition of V3H/V3V in the transverse foramen of C1 in the case mobilization and transposition of the VA are required. Multiple variants of the transcondylar approach have been described, including the atlantooccipital transarticular approach and the occipital transcondylar variant. The atlantooccipital transarticular approach involves the removal of the posterior parts of the occipital condyle and the superior articular facet of C1; the occipital transcondylar involves the removal of only the posterior end of the occipital condyle. The lateral aspect of the intracranial end of the HC can be reached with the removal of approximately the posterior third of the condyle. Further drilling of the lateral part of the posterior two-thirds of the condyle is permitted without entering the HC as the canal is directed anteriorly and laterally [17]. Drilling of hard, cortical bone surrounding the HC exposes the venous plexus of the canal, which is a landmark to stop drilling (Figure 8) [4]. This is followed by a circular dural incision around the VA, allowing the artery to be mobilized.

**Figure 9:**
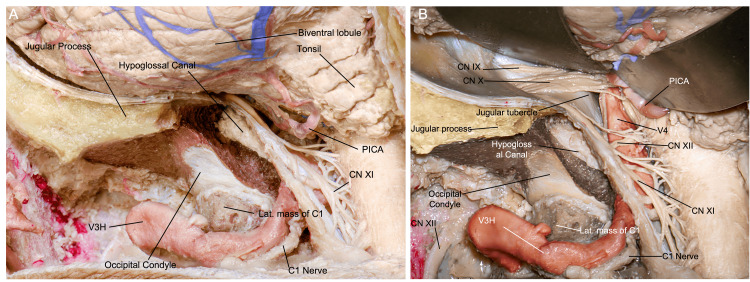
Surgical view after durotomy, where (A) we can appreciate the cerebellar hemisphere and tonsil, C1 and C2 rootlets, and (B) after mobilization of the cerebellum, the jugular foramen with the lower cranial nerves, intradural vertebral artery, and anterolateral portion of the foramen magnum. (Published with permission of the University of California San Francisco’s Skull Base & Cerebrovascular Laboratory.) CN, cranial nerve; C, cervical; PICA, posterior inferior cerebellar artery; V3H, vertebral artery horizontal segment

To gain further access to extradural lesions along the anterior and lateral margins of the foramen magnum, the condyle can be removed more extensively. Extradural removal of the JT can also improve exposure to the brainstem and mid-clivus; however, it is essential to note that CN IX, X, and XI are nearby. Ultimately, the occipital transcondylar variant is directed through the condyle and below the HC (Figure 9A) [16]. The condyle length should be noted when performing the condylectomy, as their lengths may vary and affect the amount of condyle resected. Moderate (20-24 mm) condyles are the most common (80%), with long (>24 mm) condyles being slightly more common (13%) than short (<20 mm) ones (7%). This is important surgically because an excessive condylectomy on a short condyle may result in greater CCJ instability. In contrast, a long condyle may require more extensive resection for optimal visualization. Although the FL approach has been designed to expose the CCJ, anterior FM, and LC, other corridors, such as the posterolateral incisural space, can be reached. This space extends superiorly to the level of the posteroinferior surface of the splenium and crus of the fornix and laterally to the pulvinar of the thalamus and posterior parahippocampal gyrus (Table 2) [19].

**Table 2:** FL approach modifications. AICA, anterior inferior cerebellar artery; CCJ, craniocervical junction; CN, cranial nerve; FL, far lateral; IJV, internal jugular vein; LC, lower clivus; PICA, posterior inferior cerebellar artery

Approach Name	Exposed Anatomy	Approach Process	Limits of Approach	Pathologies to be Treated
Transcondylar	CCJ, anterior foramen magnum, and lateral LC	Removal of the posterior third of the occipital condyle. May remove more of the condyle as well as the superior articular facet of C1.	Limited anteriorly by the hypoglossal canal	Lesions in the pontomesencephalic junction, anterior and lateral margins of the foramen magnum, and inferior CNs
Supracondylar	Petroclival region, inferior cranial complex, basal cisterns, and LC anterior to inferior cranial nerves, PICA, and AICA	Directed through the occipital condyle above the hypoglossal canal, involves removal of the jugular tubercle	Limited anteriorly by the petroclival junction and superolaterally by the jugular foramen	Vascular lesions involving AICA and PICA, lesions anterior to the brainstem and clivus
Paracondylar	Posterior part of the jugular foramen, mastoid process lateral to the jugular foramen, intra-extradural inferior cranial complex, IJV	Drilling of the jugular process lateral to the occipital condyle. A posterior partial mastoidectomy might be necessary.	Limited anteriorly by CN VII, IJV, external carotid, and its branches; laterally by the mastoid bone; posteriorly by vertebral artery; medially by the occipital condyle and CN XII	Lesions involving the lower cranial nerves or the jugular foramen

**Figure 10:**
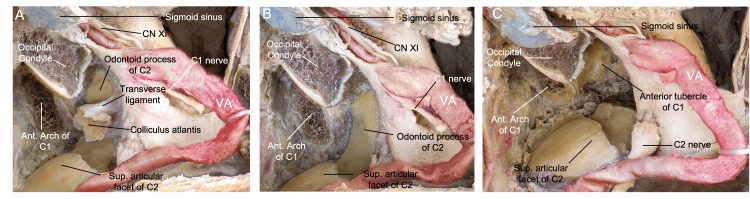
Overview of the transodontoid variation of the FL approach. In this posterolateral, surgical perspective, (A) the ipsilateral colliculus atlantis with the transverse ligament attached is shown after resection of the lateral mass of C1. (B) After removal of the colliculus and ligament, we have a direct view of the odontoid process. (C) After drilling of the odontoid process, the anterior facet for the dens and contralateral C1 is observed. (Published with permission of the University of California San Francisco’s Skull Base & Cerebrovascular Laboratory.) C, cervical; VA, vertebral artery; CN, cranial nerve; FL, far lateral

A variation of the transcondylar approach is the extreme lateral transodontoid approach, which is used to treat ventral CCJ pathology (e.g., chordomas). This approach extends the surgical corridor to the contralateral side extradurally. It involves a posteromedial mobilization of the VA after removing the posterior arch and part of the anterior arch and lateral mass of C1 to access the odontoid process and surrounding ligaments (Figure 10B). Removal of these integral structures leads to significant craniocervical instability and thus requires craniocervical fusion between the C3 and occiput, done during the same operation. The posteromedial two-thirds of the occipital condyle is drilled to expose the HC, and the JT is removed, if necessary, to expand the surgical corridor by 2-4 mm [20].

Supracondylar approach

**Figure 11:**
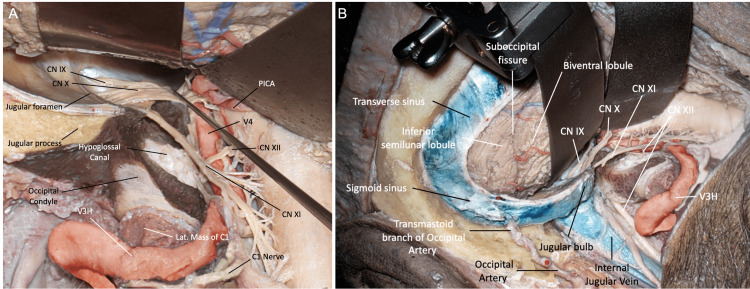
FL modifications. (A) Supracondylar modification. After removal of the jugular tubercle, the anterior medullary cistern and lower clivus can be reached. (B) Paracondylar modification. The jugular process has been drilled and the jugular bulb and exit of the lower cranial nerves can be visualized. (Published with permission of the University of California San Francisco’s Skull Base & Cerebrovascular Laboratory.) CN, cranial nerve; C, cervical; FL, far lateral; V3H, vertebral artery horizontal segment

The supracondylar approach is directed through the area superior to the HC (Figure 11A). It provides access to the superior half of the HC and the inferior portion of the jugular foramen via the JT. It can be directed through the occipital condyle above the HC or to the lateral side of the clivus. The extradural removal of the JT that blocks access to the basal cisterns and the clivus in front of the lower cranial nerves increases visualization of the area in front of the brainstem and exposes the origin of a PICA that arises from the distal VA near the midline. The JT is located above and anterior to the HC. The lateral margin of the JT is situated just medial to and below the medial edge of the jugular bulb [16]. It is located medial to the JF, between the jugular bulb and the inferior petrosal sinus. The mean length of the JT along its longitudinal axis from the posterior condylar canal to the basilar part of the clivus (parallel to the FM) is 1.6 cm. The mean width of the JT, measured along a line perpendicular to the FM from the HC aperture to the anterior infrajugular point, is 1.1 cm. The thickness of the JT, measured perpendicular to the length and width at the level of the HC, is 0.6 cm.

Drilling of the JT is challenging, and significant injury to neurovascular structures in the region can occur if the anatomy is not clearly localized. Drilling occurs from the HC toward the clivus along the length of the JT. Removal of the JT greatly increases the exposure of key anatomical structures in the petroclival region. Under a cadaver simulation setup, it increases the exposed length of CN XI and the anterior inferior cerebellar artery (AICA) by 9.3 and 8.0 mm, respectively. Drilling the JT also exposes 15.6 mm of the basilar artery [21]. During the drilling of the JT, surgeons will likely encounter copious bleeding related to the posterior condylar emissary veins. CT may enable preoperative prediction of the origin and course of the vein to guide the surgeon in choosing the direction in which to drill to avoid disruption of the sinuses. Caution is also required in drilling the JT to avoid damaging the lower cranial nerves (Table 2) [4].

Paracondylar approach

The paracondylar approach includes drilling the jugular process of the occipital bone in the area lateral to the posterior half of the condyle (Figure 11B). It provides access to the posterior part of the JF, the posterior aspect of the facial nerve (CN VII), and the mastoid process on the lateral side of the JF. The exposure of the posterior surface of the jugular bulb can also be extended laterally into the posterior aspect of the mastoid to access the mastoid segment of CN VII and the stylomastoid foramen [16]. A pivotal structure for this approach is the jugular process, which is located posterior to the jugular foramen and serves as an attachment point for the rectus capitis lateralis [22]. The jugular process extends laterally from the posterior half of the occipital condyle. The occipitomastoid suture passes between the lateral edge of the jugular process and the medial edge of the digastric groove, the orifice of the facial canal, and the styloid process. Medial and superficial, the posterior belly digastric muscle covers the rectus capitis lateralis and jugular process. Thus, removing the mastoid tip and digastric muscle exposes the insertion of the rectus capitis lateralis into the inferior surface of the jugular process [22]. Removing the rectus capitis lateralis muscle and drilling the jugular process of the occipital bone can significantly increase the posterior exposure around the JF. Importantly, the jugular process is surrounded by two condylar emissary veins, the lateral and posterior condylar veins. The lateral condylar vein branches from the anterior condylar confluence coursing along the lateral surface of the occipital condyle and draining into the vertebral venous plexus. The posterior condylar vein courses into the posterior condylar canal and connects the suboccipital venous plexus with the sigmoid sinus or jugular bulb. The suboccipital venous plexus subsequently drains into the deep cervical vein and anterior vertebral vein. Preoperative examination of the courses of these veins is critical to avoid unnecessary bleeding and embolism during surgery. The anatomical limits of the region accessed by this approach include the carotid canal anteriorly, the mastoid air cells laterally, the VA posteriorly, and the occipital condyle and CN XII medially [22]. This approach can be combined with extreme lateral extension, which extends the approach laterally through the mastoid tip. According to the inferomedial exposure required to maneuver within the jugular region, medial mobilization of the VA after opening the transverse foramen of C1 might be needed (Table 2).

## Conclusions

Understanding the spatial relationship between the anatomical structures and the relevance of the occipital condyle in the FL approach and its modifications is of the utmost importance. The FL approach provides ample exposure to the LC and the suboccipital area. This study incorporated interactive VMs to facilitate the understanding of the neuroanatomical structures and exposure provided by the FL approach and its modifications. Although anatomical variations are discussed in the text, they are not incorporated into the figures and VMs, which serves as a limitation of this study. Regardless, understanding the foundational anatomy can help contextualize any variations that may arise during surgery. We hope that the technology used in this paper can be used for anatomical teaching and to describe other complex procedures in neurosurgery. We also hope that clinicians and surgeons in other fields can adopt this technology to improve teaching and understanding of complex anatomy throughout the human body.

## References

[1] Hammon WM, Kempe LG (1972). The posterior fossa approach to aneurysms of the vertebral and basilar arteries. J Neurosurg.

[2] Heros RC (1986). Lateral suboccipital approach for vertebral and vertebrobasilar artery lesions. J Neurosurg.

[3] Wang M, Chae R, Shehata J (2019). Comparative analysis of the subtonsillar, far-lateral, extreme-lateral, and endoscopic far-medial approaches to the lower clivus: an anatomical cadaver study. World Neurosurg.

[4] Rhoton AL Jr (2000). The far-lateral approach and its transcondylar, supracondylar, and paracondylar extensions. Neurosurgery.

[5] Vigo V, Hirpara A, Yassin M (2020). Immersive surgical anatomy of the craniocervical junction. Cureus.

[6] Rubio RR, Shehata J, Kournoutas I (2019). Construction of neuroanatomical volumetric models using 3D scanning techniques: Technical note and applications. World Neurosurg.

[7] El-Bary THA, Dujovny M, Ausman JI (1995). Microsurgical anatomy of the atlantal part of the vertebral artery. Surg Neurol.

[8] Alvernia JE, Fraser K, Lanzino G (2006). The occipital artery: a microanatomical study. Neurosurgery.

[9] Martins C, Yasuda A, Campero A, Ulm AJ, Tanriover N, Rhoton A Jr (2005). Microsurgical anatomy of the dural arteries. Neurosurgery.

[10] Parkinson D (1997). The suboccipital cavernous sinus. J Neurosurg.

[11] Chaddad F, Doria-Netto HL, Campos JM de (2014). The far-lateral craniotomy: tips and tricks. Arq Neuropsiquiatr.

[12] Lawton MT (2011). Seven Aneurysms, 1st ed. https://doi.org/10.1055/b-002-66278.

[13] Campero A, Villalonga JF, Elizalde RL, Ajler P (2018). The nuchal lines as anatomical landmarks to dissect the muscles in the far-lateral approach. World Neurosurg.

[14] Sato A, Hirai S, Obata Y, Maehara T, Aoyagi M (2018). Muscular-stage dissection during far lateral approach and its transcondylar extension. J Neurol Surg B Skull Base.

[15] Ulm AJ, Quiroga M, Russo A, Russo VM, Graziano F, Velasquez A, Albanese E (2010). Normal anatomical variations of the V₃ segment of the vertebral artery: surgical implications. J Neurosurg Spine.

[16] Katsuta T, Rhoton AL Jr, Matsushima T (1997). The jugular foramen: microsurgical anatomy and operative approaches. Neurosurgery.

[17] Paraskevas GK, Tsitsopoulos PP, Papaziogas B (2009). Osseous variations of the hypoglossal canal area. Medical Science Monitor : International Medical Journal of Experimental and Clinical Research.

[18] Verma R, Kumar S, Rai AM, Mansoor I, Mehra RD (2016). The anatomical perspective of human occipital condyle in relation to the hypoglossal canal, condylar canal, and jugular foramen and its surgical significance. J Craniovertebr Junction Spine.

[19] Giammattei L, Starnoni D, Benes V (2021). Extreme lateral supra cerebellar infratentorial approach: surgical anatomy and review of the literature. World Neurosurg.

[20] Alzhrani G, Gozal YM, Eli I, Sivakumar W, Raheja A, Brockmeyer DL, Couldwell WT (2018). Extreme lateral transodontoid approach to the ventral craniocervical junction: cadaveric dissection and case illustrations. J Neurosurg.

[21] Zhang H, Lan Q, Wang X (2011). Neuronavigation-based quantitative study of the far-lateral keyhole approach following partial removal of the occipital condyle and jugular tubercle. J Clin Neurosci.

[22] Komune N, Matsuo S, Miki K (2019). Microsurgical anatomy of the jugular process as an anatomical landmark to access the jugular foramen: A cadaveric and radiological study. Oper Neurosurg (Hagerstown).

